# Isolation and Identification of the Indigenous Yeast Population during Spontaneous Fermentation of Isabella (*Vitis labrusca* L.) Grape Must

**DOI:** 10.3389/fmicb.2017.00532

**Published:** 2017-03-29

**Authors:** María L. Raymond Eder, Cristina Reynoso, Santiago C. Lauret, Alberto L. Rosa

**Affiliations:** ^1^Laboratorio de Genética y Biología Molecular, IRNASUS–Consejo Nacional de Investigaciones Científicas y Técnicas, Facultad de Ciencias Químicas, Universidad Católica de CórdobaCórdoba, Argentina; ^2^Bodega La CaroyenseColonia Caroya, Argentina

**Keywords:** *Vitis labrusca* L., Isabella, grapes, spontaneous fermentation, wine, microbiota, yeast

## Abstract

Grape must harbors a complex community of yeast species responsible for spontaneous alcoholic fermentation. Although there are detailed studies on the microbiota of *Vitis vinifera* L. grapes, less is known about the diversity and behavior of yeast communities present on fermenting grape must from other species of *Vitis*. In this work, we used a culture-dependent method to study the identity and dynamics of the indigenous yeast population present during the spontaneous fermentation of Isabella (*Vitis labrusca* L.) grape must. Alcoholic fermentation was conducted using standard enological practices, and the associated non-*Saccharomyces* and *S. cerevisiae* yeast community was analyzed using selective growth media and 5.8-ITS DNA sequencing. *Candida californica, Candida hellenica, Starmerella bacillaris* (synonym *Candida zemplinina*), *Hanseniaspora uvarum*, and *Hanseniaspora vineae* were the main non-*Saccharomyces* species identified on Isabella fermenting must. *Issatchenkia hanoiensis*, a yeast species rarely found on *Vitis vinifera* L. grapes, was also recognized on Isabella grape must. *Candida azymoides, Candida californica* and *Pichia cecembensis*, identified in this work on Isabella fermenting must, have not previously been found on *Vitis vinifera* L. grape must. Interestingly, *C. azymoides, I. hanoiensis* and *P. cecembensis* have recently been isolated from the surface of *Vitis labrusca* L. grapes from vineyards in the Azores archipelago, suggesting that specific *Vitis*-yeast species associations are formed independently of geographic origin. We suggest that *C. azymoides, C. californica*, and *P. cecembensis* are yeast species preferentially associated with *Vitis labrusca* L. grapes. Specific biological interactions between grapevines and yeast species may underlie the assembly of differential *Vitis*-microbial communities.

## Introduction

The surface of grapes lodges the microbiota responsible for spontaneous fermentation of grape must into wine (Mortimer and Polsinelli, 1999; Barata et al., 2012). The complexity of indigenous non-*Saccharomyces* and *Saccharomyces* yeast microbiota present during spontaneous grape must fermentation has a major impact on the organoleptic and sensory properties of the final wines (Jolly et al., 2013; Padilla et al., 2016b; Varela and Borneman, 2016).

The identity and relative abundance of indigenous yeast species on grapes is considered to be dependent on the *terroir* (i.e., soil type, annual mean temperature, and rainfall, etc.), the ripeness and health of the grapes, as well as the production procedures in the vineyards (Mortimer and Polsinelli, 1999; Bokulich et al., 2014; Drumonde-Neves et al., 2016; Grangeteau et al., 2016). The resulting grapevine microbiota could specifically identify a vineyard and, accordingly, the identity of wines from that particular *terroir* (Bokulich et al., 2014; Knight et al., 2015; Capece et al., 2016).

Detailed studies on the microbiota of grapes and grape musts may enable valuable indigenous yeast strains that contribute to the regional character of wines to be identified (Knight et al., 2015; Capece et al., 2016; Padilla et al., 2016b; Varela and Borneman, 2016). Molecular methods permit rapid and precise identification of yeasts at the species or strain level (Barata et al., 2012). Among these, the sequencing of rDNA regions D1–D2 (James et al., 1997; Kurtzman and Robnett, 1998), RFLP analyses (Guillamón et al., 1998) and/or the sequencing of ribosomal 5.8S-ITS are frequently used for rapid identification of indigenous isolated yeasts (Barata et al., 2012).

Non-*Saccharomyces* are the predominant yeast species isolated at early stages of the spontaneous fermentation of *Vitis vinifera* L. grape musts (Combina et al., 2005; Jolly et al., 2013; Padilla et al., 2016b). Among these, *Hanseniaspora, Candida, Pichia*, and *Metschnikowia* are the most important genera (Jolly et al., 2013; Varela and Borneman, 2016). As fermentation progresses, the population of non-*Saccharomyces* species decreases and the wine yeast *Saccharomyces cerevisiae* completes the fermentation process (Albergaria and Arneborg, 2016). The ability of *S. cerevisiae* to outcompete non-*Saccharomyces* species is associated with its higher fermentative power as well as its additional advantageous phenotypes that include alcohol tolerance and the secretion of killer-like compounds (Albergaria and Arneborg, 2016). Although there are detailed studies on yeast microbiota from *Vitis vinifera* L. grape musts (Combina et al., 2005; Zott et al., 2008; Albertin et al., 2014; Padilla et al., 2016a), less is known about the communities of yeast present on grapes from other species of *Vitis*. Moreover, the potential existence of selective interactions between various grapevine and microbial species, which may underlie the assembly of specific microbial communities (Wolfe and Dutton, 2015), is unknown.

Isabella grapes are one of the main varieties of *Vitis labrusca* L., a plant originally from the south of the United States, with worldwide distribution (Hernandez et al., 2011; Hurrel et al., 2014; Almanza-Merchán et al., 2015). We hypothesized that the microbial community of Isabella grape must, as well as other poorly explored ecological niches, constitutes a potential source of relevant yeast strains for the winemaking industry. We report here a study of the yeast microbiota present during spontaneous fermentation of Isabella grape must from Colonia Caroya, Argentina.

The Isabella vineyards of Colonia Caroya have remarkable historical value because they are located in a traditional winemaking region in Córdoba, Argentina, initiated four centuries ago by the Jesuit missions. Cultivated grapes in Colonia Caroya are mostly of *Vitis vinifera* L. (i.e., ∼75% of the vineyards) and Isabella grapes are mainly used for making grape juice. Some local producers and wineries, however, still culture and harvest these grapes to prepare either pure Isabella or Isabella blended wines. Our study is the first analysis of indigenous yeasts on grapes from a *Vitis* species different from *Vitis vinifera* L. in Argentina. It is also the first characterization of the indigenous yeasts in fermenting grape must from Córdoba, an emerging winemaking region in Argentina.

## Materials and Methods

### Spontaneous Fermentation of Isabella Must

Isabella grapes were harvested in Colonia Caroya vineyards (vintage of March 2015), located at 31°02′00″S/64°05′36″O and 491 m above sea level, in the province of Córdoba, Argentina. The region has an annual rainfall of 765 mm and a mean temperature of 15.8°C. Spontaneous fermentation of destemmed and partially crushed Isabella grapes (supplemented with 85 mg/l sodium metabisulfite) was performed in a reference local winery. A must sample of ∼60 lt (an aliquot taken from a total of 15,000 kg of Isabella grapes subjected to spontaneous fermentation) was fermented at 25–28°C in a stainless steel tank located in a room of the winery not previously used for winemaking. Isabella must in the stainless steel tank was punched down twice a day. Aliquots from the must were taken daily for 5 days (i.e., 0, 24, 48, 72, and 96 h) and stored in 30% (v/v) glycerol at -70°C.

### Yeast Isolation, Culturing, and Characterization

Appropriate dilutions of fermenting Isabella must samples (i.e., *t0, t24, t48, t72*, and *t96*) were plated in duplicate on both YPD-Cm agar [yeast extract 1% (w/v), peptone 2% (w/v), glucose 2% (w/v), agar 2% (w/v), chloramphenicol 10 μg/ml] and YPD-Cm-Cx agar [yeast extract 1% (w/v), peptone 2% (w/v), glucose 2% (w/v), agar 2% (w/v), chloramphenicol 10 μg/ml, cycloheximide 0.5 μg/ml]. Plates were incubated for 5 days at 25°C. Colony counts on YPD-Cm and YPD-Cm-Cx plates were used to determine the relative contribution of total yeasts versus non-*Saccharomyces* (i.e., cycloheximide resistant) yeast species, respectively (Zott et al., 2008).

For these studies, we first determined the concentration of cycloheximide able to inhibit the growth of reference *Saccharomyces* spp. strains (i.e., *Saccharomyces bayanus* – strain CIVC 8130 – and *S. cerevisiae* –strain EC1118), while still allowing the growth of reference non-*Saccharomyces* strains (i.e., *Torulaspora delbrueckii* –strain RG07–, *Metschnikowia pulcherrima* –strain RG01–, *Pichia membranifaciens* –strain RG02– and *Hanseniaspora uvarum* –strain RG06–). Non-*Saccharomyces* strains, RG07, RG01, RG02 and RG06, were kindly provided by R. Gonzalez (ICVV; Logroño, Spain). Strains were plated (i.e., ∼50 viable cells) on YPD-Cm agar supplemented with various concentrations of cycloheximide (i.e., between 0 and 4.0 ug/ml). Plates were incubated during 5 days at 25°C and growth was considered positive when colonies larger than 0.25 ± 0.05 mm were observed. Based on the sensitivity of the reference strains to cycloheximide, YPD-Cm agar supplemented with cycloheximide 0.5 μg/ml was used in subsequent studies. Isabella must samples *t0, t24, t48, t72*, and *t96* were also plated in duplicate on WL-Cm Nutrient agar [Oxoid WL Nutrient agar medium 7.5% (w/v), chloramphenicol 10 μg/ml] and incubated for 5 days at 25°C. WL-Cm Nutrient agar was used to recognize alternative colony phenotypes potentially corresponding to different yeast species.

Forty randomly selected yeast colonies were isolated from YPD-Cm agar plates from each sampling time. These colonies enabled strains to be identified representing ≥2.5% of the yeast species present at each sampling time. To assure random isolation, plates (i.e., YPD-Cm agar) with 30–50 independent yeast colonies were superimposed on a design with 20-evenly separated dots. The 20 yeast colonies closest to each dot were selected from two independent YPD-Cm agar plates. In addition, 10 yeast colonies showing different phenotypes (i.e., morphology and/or color) were isolated from WL-Cm Nutrient agar plates. These colonies could correspond to rare yeast species present at each sampling. All isolated yeast strains were streaked on YPD agar, grown for 48 h at 25°C, and stored at -70°C in YPD broth with 30% (v/v) glycerol added. A total of 250 yeast strains were isolated from the five samplings of spontaneously fermenting Isabella grape must.

### Molecular Identification of Yeast Species

All isolated strains (i.e., 250) were identified by PCR amplification and sequencing of their 5.8-ITS (*Internal Transcribed Spacer*) rDNA regions (Granchi et al., 1999), using ITS1 and ITS4 primers (White et al., 1990). Total genomic DNA was extracted from 4.5 ml yeast cultures grown in YPD broth with orbital shaking (200 rpm) during 48 h at 25°C. Cells were collected by centrifugation at 3,000 rpm for 30 s in a bench top centrifuge and washed with sterile distilled water. Yeasts were disrupted in 400 μl of buffer TENT [HCl-Tris 10 mM (pH 8.0), Na-EDTA 1 mM, NaCl 100 mM, Triton X-100 2% (v/v) and SDS 1% (w/v)] using 0.1 ml of acid-washed glass beads (Scientific Industries, Inc.) and a Disruptor Genie (Scientific Industries, Inc.) apparatus. Broken cells had 50 μl of cold sodium acetate 5 M (pH 4.7) added and were centrifuged for 10 min at 10,000 rpm. Supernatants (i.e., 300–350 μl) were carefully transferred into clean tubes and supplemented with 300 μl of cold isopropanol. DNA was recovered by 10 min centrifugation at 10,000 rpm, washed with 1.0 ml of 70% (v/v) ice-cold ethanol and re-centrifuged at 10,000 rpm for 5 min. Pelleted DNA was dried at room temperature and suspended in 50 μl of buffer TE. PCR mixtures contained 100 ng DNA, 1.5 mM MgCl_2_, *Taq* polymerase buffer 1X (Invitrogen, USA), 200 μM dNTPs, 10 pmol of each ITS1 and ITS4 and 1.25 units of *Taq* polymerase (Invitrogen, USA). The amplification reaction was performed in a MJ Mini Bio-Rad thermocycler (Bio-Rad, USA) using an initial denaturation step at 93°C for 3 min, followed by 35 cycles of: 93°C for 30 s, annealing at 52°C for 30 s, extension at 72°C for 1 min followed by a final extension at 72°C for 10 min. PCR products were subjected to DNA sequencing (Macrogen, Korea) and the resulting sequences were analyzed using BLASTN software and GenBank NCBI reference sequences. Species identification was considered valid when the identity of a 5.8-ITS sequence and a reference sequence was ≥98%. Sequences from representative strains were deposited in the GenBank NCBI database with the accession numbers *KY693700* (*Candida azymoides*: IT0-016), *KY693709* (*Candida californica*: IT2-010), *KY693702* (*H. uvarum*: IT0-031), *KY693711* (*Hanseniaspora vineae*: IT2-021), *KY693701* (*Issatchenkia hanoiensis*: IT0-025), *KY693704* (*Pichia cecembensis*: IT0-042), *KY693703* (*Starmerella bacillaris*: IT0-033), *KY693705* (*S. bacillaris*: IT1-027), *KY693706* (*S. bacillaris*: IT1-033), *KY693708* (*S. cerevisiae*: IT2-031), *KY693710* (*S. cerevisiae*: IT4-007), and *KY693707* (*T. delbrueckii*: IT1-039).

### Killer Phenotype and Ethanol Tolerance

The killer phenotype was analyzed using the diffusion agar technique (Lopes and Sangorrín, 2010) on YPD-methylene blue (MB) medium [i.e., yeast extract 1% (w/v), peptone 2% (w/v), glucose 2% (w/v), agar 2% (w/v), 0.003% (w/v) methylene blue] buffered at pH 4.6 with 0.1 M citrate-phosphate buffer (Lopes and Sangorrín, 2010). Potential killer yeast strains were grown on YPD broth and spotted (i.e., 3 μl 10^6^ cells/ml) onto a lawn of each tested killer-sensitive strain. Lawns were prepared using molten YPD-MB agar inoculated with 10^5^ cells/ml. Plates were incubated for 72 h at 24°C. *S. cerevisiae* CLIB154 (K2 killer) and *S. cerevisiae* BY4742 (K2 sensitive) were used as reference strains. Strains CLIB154 and BY4742 were kindly provided by M. Bely (ISVV; Bordeaux, France).

To study ethanol tolerance, we developed a simple spot assay on low dextrose [i.e., glucose 0.5% (w/v)] YPD agar supplemented with either 0, 2.5, 5.0, 7.5, or 10.0% (v/v) ethanol. The test was validated using *S. cerevisiae* strain EC1118. In these studies, strains were grown overnight at 25°C on YPD broth and cells (i.e., 3 μl of a suspension of 10^6^ cells/ml) were spotted onto the agar. Growth was observed after 48 h at 22°C and considered positive when colonies larger than 0.25 ± 0.05 mm were visible. The following Isabella strains isolated in this work were analyzed: *C. californica* (strain IT2-010), *H. vineae* (strain IT2-021), *P. cecembensis* (strain IT0-042), *S. cerevisiae* (strains IT2-031 and IT4-007), *S. bacillaris* (strains IT0-033, IT1-027, and IT1-033), and *T. delbrueckii* (strain IT1-039).

## Results

### Dynamics of Non-*Saccharomyces* and *S. cerevisiae* Yeast Populations

**FIGURE 1:**
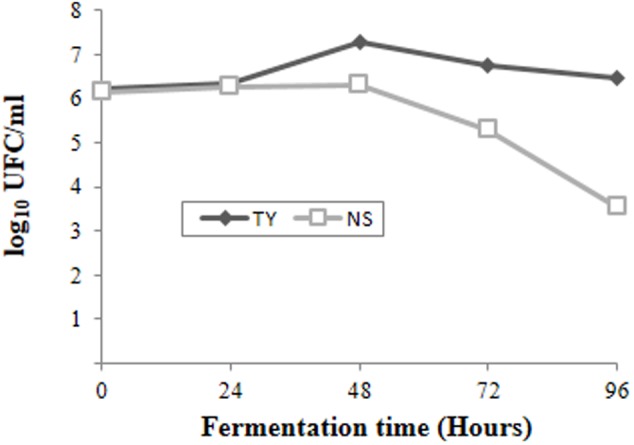
**Population dynamics of non-*Saccharomyces* (NS) and total (TY) yeasts at different times during spontaneous fermentation of Isabella grape must**.

The spontaneous fermentation of Isabella grape must was almost complete in 5 days (i.e., *t96*). Results from physicochemical analyses of the must are shown in **Table 1**. After 5 days of fermentation, the concentration of the remaining reducing sugars in the Isabella must was 3.47 g/l. A final concentration of 8.2% ethanol was achieved (**Table 1**). The final volatile acidity and pH were 0.30 g/l and 3.38, respectively. For yeast population analyses, samples from the Isabella fermenting must were taken every 24 h, from *t0* to *t96* (see Materials and Methods). The relative contribution of non-*Saccharomyces* and *S. cerevisiae* yeasts, among the total yeast population, was determined on YPD-Cm agar media supplemented or not with cycloheximide. **Table 2** shows that YPD-agar containing 0.5 ug/ml cycloheximide was able to inhibit the growth of reference *Saccharomyces* spp. strains, still allowing the growth of reference non-*Saccharomyces* strains. **Figure 1** shows that non-*Saccharomyces* were the predominant yeast species at early stages of Isabella grape must fermentation (i.e., *t0* and *t24*). In the mid-stages of fermentation (*t48* and *t72*), the total yeast count increased, with *S. cerevisiae* being the prevalent species (**Figure 1**). The complete dominance of *S. cerevisiae* was evident after 5 days of fermentation (*t96*) when only ≤10^4^ colonies of non-*Saccharomyces* species developed on YPD-Cm-Cx media (**Figure 1**). The observed dynamics of the non-*Saccharomyces* and *S. cerevisiae* yeast populations are similar to those observed on spontaneously fermenting must of *Vitis vinifera* L. grapes (Combina et al., 2005; Zott et al., 2008; Jolly et al., 2013).

**Table 1:** Physicochemical analyses of Isabella grape must.

	Fermentation (hours)	
	0	96
Density (g/ml; 20°C)	1.067	nd
Ethanol (g/100 ml)	0	8.20
Brix (degrees)	16	nd
Reducing sugars (g/l)	150	3.47
Total acidity (tartaric acid) (g/l)	7.55	7.05
Volatile acidity (acetic acid) (g/l)	0.29	0.30
Total SO_2_ (mg/l)	83	55
pH	3.40	3.38

*nd, not determined.*

**Table 2:** Resistance of reference yeasts to cycloheximide.

Species	Strain	Cycloheximide (μg/ml)					
		0	0.5	1.0	2.0	3.0	4.0
*Torulaspora delbrueckii*	RG07	+	+	-	-	-	-
*Hanseniaspora uvarum*	RG06	+	+	+	+	-	-
*Pichia membranifaciens*	RG02	+	+	-	-	-	-
*Metschnikowia pulcherrima*	RG01	+	+	+	+	+	+
*Saccharomyces bayanus*	CIVC 8130	+	-	-	-	-	-
*Saccharomyces cerevisiae*	EC1118	+	-	-	-	-	-

*+, growth; -, no growth.*

### Identification of Yeast Species of Isabella Fermenting Grape Must

**FIGURE 2:**
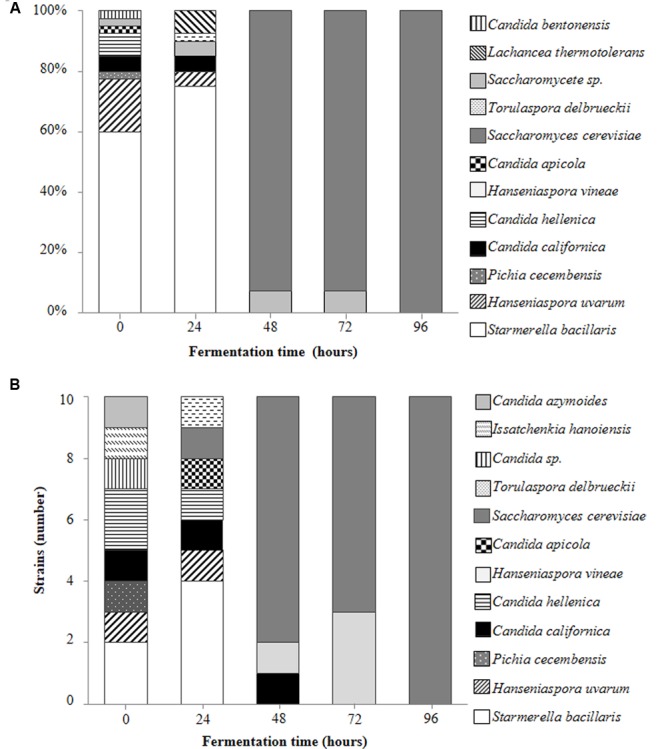
**Main contributing yeast species during spontaneous fermentation of Isabella grape must**. Percentages represent the relative contribution of the indicated yeast species among 40 randomly selected colonies obtained at the indicated times of fermentation **(A)**. Yeast species identified among 10 rare colonies isolated from WL-Cm Nutrient agar plates at the indicated times of fermentation **(B)**.

A total of 250 yeast strains were isolated and identified from the fermenting Isabella grape must (see Materials and Methods). These various yeast species are shown in **Figures 2A,B**. A wide diversity of non-*Saccharomyces* yeasts was observed at early stages of fermentation (**Figures 2A,B**; *t0* and *t24*), with *S. bacillaris, H. uvarum*, and *Candida hellenica* being the major species at *t0* (62.5, 17.5, and 7.5% of the total yeast population, respectively) (**Figure 2A**). *C. californica* was observed among colonies isolated from both YPD-Cm agar and WL-Cm Nutrient agar plates (**Figures 2A,B**). *C. azymoides, C. bentonensis, I. hanoiensis*, and *P. cecembensis*, rarely recognized in winemaking ecosystems, were isolated early in fermentation. *S. bacillaris* was the most abundant species (i.e., 75%) at *t24* (**Figure 2A**). *S. cerevisiae*, although present at very low percentages during early stages of fermentation, was able to dominate the yeast population as from *t48* (**Figures 1**, **2A**). This result was expected, based on the well-known predominance of *S. cerevisiae* at intermediate and advanced stages of the spontaneous fermentation of grape musts from various origins (Fleet, 2003). *H. vineae* was the main non-*Saccharomyces* species coexisting with *S. cerevisiae* at advanced times of Isabella grape must fermentation (i.e., *t48* and *t72*). No non-*Saccharomyces* yeast species were observed among the 40 random isolated colonies at *t96* (**Figure 2A**). Moreover, all the 10 yeast colonies selected at *t96* on WL Nutrient medium, based on their rare morphological and/or color phenotypes, corresponded to *S. cerevisiae* strains (**Figure 2B**).

### Killer Sensitivity and Ethanol Tolerance of *S. bacillaris* Isolates

Based on the relative contribution of *S. bacillaris* at *t0* and *t24*, we expected to find at least ∼10% of *S. bacillaris* colonies at *t48*. This species, however, was not represented among the 50 yeast strains isolated at *t48* (**Figures 2A,B**). We hypothesized that this may result from the sensitivity of this species to unknown indigenous killer strains developing in the interval *t24*–*t48*. A similar phenomenon has been described for other non-*Saccharomyces* species present in mixed fermentations (Pérez-Nevado et al., 2006; de Ullivarri et al., 2014). To study this possibility, isolated *S. bacillaris* strains were used as lawn in a standard killer-assay (see Materials and Methods). No particular sensitivity of the *S. bacillaris* isolates was observed when challenged to different non-*Saccharomyces* or *S. cerevisiae* isolates as from *t48* (not illustrated). We also considered the possibility that the decrease in the population of *S. bacillaris* at *t48* was based on a low tolerance of these strains to ethanol. *S. bacillaris* strains have been described as tolerant to high levels of ethanol (i.e., 8–12%) (Combina et al., 2005; Tofalo et al., 2011). **Table 3** shows that *S. bacillaris* strains isolated from Isabella fermenting must developed only up to 5% alcohol while other isolated non-*Saccharomyces* strains tolerated higher levels of ethanol (i.e., 7.5%). This low tolerance to ethanol could explain, at least in part, why *S. bacillaris* was not isolated at *t48*, when the ethanol concentration was 4–5% (not shown). These results indicate that ethanol tolerance may dramatically differ between *S. bacillaris* strains isolated from various geographic origins and/or *Vitis* species. It was also remarkable from these studies that indigenous Isabella *S. cerevisiae* strains (**Table 3**) had relatively low tolerance to ethanol (i.e., <10%) compared with indigenous *S. cerevisiae* isolates from *Vitis vinifera* L. (Baǧder Elmac et al., 2014; Capece et al., 2016; Šuranská et al., 2016).

**Table 3:** Ethanol tolerance of yeast species isolated from Isabella grape must.

Species	Strain	Ethanol (%)				
		0	2.5	5.0	7.5	10.0
*Candida californica*	*IT2*-010	+	+	+	+	-
*Starmerella bacillaris*	*IT0*-033	+	+	+	-	-
*Starmerella bacillaris*	*IT1*-027	+	+	+	-	-
*Starmerella bacillaris*	*IT1*-033	+	+	+	-	-
*Hanseniaspora vineae*	*IT2*-021	+	+	+	+	-
*Pichia cecembensis*	*IT0*-042	+	+	+	+	-
*Saccharomyces cerevisiae t48*	*IT2*-031	+	+	+	+	-
*Saccharomyces cerevisiae t96*	*IT4*-007	+	+	+	+	-
*Torulaspora delbrueckii*	*IT1*-039	+	+	+	+	+
*Saccharomyces cerevisiae*	EC1118	+	+	+	+	+

*+, growth; -, no growth.*

### Specific Yeast Microbial Communities of *Vitis labrusca* L. Grapes

Non-*Saccharomyces* species recognized on fermenting Isabella must include a large diversity of yeasts (**Figures 2A,B**). Among these, we isolated *I. hanoiensis*, which is rarely found in winemaking environments (Hierro et al., 2006). *I. hanoiensis* has also been recognized in a recent elegant and comprehensive study of the indigenous yeast community present at the surface of *Vitis labrusca* (L. cultivars and its hybrids) grapes from the Azores archipelago (Drumonde-Neves et al., 2016). We also found *C. azymoides, C. californica* and *P. cecembensis* on Isabella grape must, which have not been recognized on *Vitis vinifera* L. grapes. Interestingly, *C. azymoides* and *P. cecembensis* have also been found on *Vitis labrusca* (L. cultivars and its hybrids) grapes from the Azores archipelago (Drumonde-Neves et al., 2016). It is remarkable that these two yeast species are present on *Vitis labrusca* L. grapes from two such distant regions in the world (i.e., Azores archipelago and Argentina), corresponding to different geographic regions and production conditions in their respective vineyards. Moreover, *C. azymoides* and *P. cecembensis* were not found in grape musts from *Vitis vinifera* L. vineyards in the Azores archipelago (Drumonde-Neves et al., 2017). In our study, we did not find *Barnettozyma californica*, an additional potential specific yeast from *Vitis labrusca* L. grapes (Drumonde-Neves et al., 2016). Taken together, the study of Drumonde-Neves et al. (2016) and the results reported here strongly suggest that at least *C. azymoides* and *P. cecembensis* are preferentially associated with the microbiota of *Vitis labrusca* L. grapes. To our knowledge, these observations constitute the first data about specific associations between *Vitis* and yeast species. This finding may have significant ecological and/or evolutionary interest as well as for the understanding of the spontaneous assembly of the *Vitis* microbiota.

## Discussion

Many yeast species have been recognized in spontaneously fermenting must from *Vitis vinifera* L. grapes (Combina et al., 2005; Zott et al., 2008; Aponte and Bialotta, 2016; Padilla et al., 2016a). Interestingly, the yeast microbiota of Isabella grape must reported here, as well as recently elsewhere (Drumonde-Neves et al., 2016), show a remarkable diversity of non-*Saccharomyces* yeast species. In *Vitis vinifera* L. grapes, the more relevant non-*Saccharomyces* species include the apiculate yeasts *Hanseniaspora* and a large proportion of yeasts from the *Metschnikowia* and *Candida* genera (Garofalo et al., 2015; Albergaria and Arneborg, 2016). *H. uvarum*, present at later stages during fermentation of *Vitis vinifera* L. grape must (Zott et al., 2008; Padilla et al., 2016a), was also found in spontaneously fermenting Isabella (Baffi et al., 2010; Bezerra-Bussoli et al., 2013; Hong and Park, 2013). Isabella *H. uvarum*, however, markedly decreased at the mid-stages of fermentation. In fact, *H. vineae* was the only non-*Saccharomyces* species able to coexist with *S. cerevisiae* until relatively advanced stages of fermentation of Isabella grape must.

The main non-*Saccharomyces* species contributing to the initial stages of Isabella must fermentation was *S. bacillaris*. This represented 62.5% of the strains isolated at *t0*, increasing to 75% of the total yeast population at *t24*, and it was not isolated at *t48*. This behavior was unexpected, as *S. bacillaris* appears to develop similarly to other non-*Saccharomyces* yeasts in mixed fermentations of *Vitis vinifera* L. musts (Wang et al., 2016). Moreover, similar studies identified *S. bacillaris* at advanced stages of fermentation coexisting with *S. cerevisiae* (Zott et al., 2008; Tofalo et al., 2011; Padilla et al., 2016a). Indigenous *S. bacillaris* and *S. cerevisiae* strains isolated from Isabella must showed a relatively low tolerance to ethanol. Considering the relatively low average levels of total reducing sugars on Isabella grapes from Colonia Caroya, these findings could highlight a low selective pressure for ethanol tolerance on yeast communities in these vineyards. In the case of *S. bacillaris*, however, it has been suggested that this species is not under selective pressure in winemaking environments (Masneuf-Pomarede et al., 2015).

Fourteen different non-*Saccharomyces* yeast species were identified at initial stages of spontaneous fermentation of Isabella must. Although some of these yeast species have been described in winemaking ecosystems (i.e., *H. uvarum, S. bacillaris, T. delbrueckii, H. vineae, I. hanoiensis*) (Zott et al., 2008; Albertin et al., 2014; Padilla et al., 2016a; Drumonde-Neves et al., 2017), other species such as *C. azymoides, C. californica* and *P. cecembensis*, have not previously been isolated from *Vitis vinifera* L. grapes. This observation reinforces the interest in searching for wine yeast diversity in ecological niches alternative to the traditional *Vitis vinifera* L. environments (Combina et al., 2005; Knight et al., 2015; Capece et al., 2016; Vigentini et al., 2016). For example, valuable indigenous *H. uvarum* strains, isolated from *Vitis Labrusca* (cultivar Campbell Early) grape must, have proved to be useful starters for winemaking (Hong and Park, 2013).

Interestingly, *C. azymoides* and *P. cecembensis* have recently been recognized on the surface of *Vitis labrusca* L. grapes from vineyards in the Azores archipelago (Drumonde-Neves et al., 2016). This could be explained by the fortuitous presence of rare yeast species on *Vitis labrusca* L. grapes from the Azores Archipelago, appearing because of particular environmental influences and/or vineyard and/or winery production conditions. *C. azymoides* and *P. cecembensis*, however, were not present on *Vitis vinifera* L. grape musts from the same geographic location (Azores Archipelago) (Drumonde-Neves et al., 2017). Moreover, the finding of *C. azymoides* and *P. cecembensis* during spontaneous fermentation of Isabella grapes harvested in Argentina strongly suggests that at least these two yeast species are associated with *Vitis labrusca* L. grapes, independently of their geographic origin and/or the associated human interventions. We propose that *C. azymoides, C. californica* and *P. cecembensis* are yeast species preferentially associated with *Vitis labrusca* L. grapes. Specific *Vitis*-microbial interactions may underlie the assembly of specific grapevine yeast communities. Modern metagenomic approaches (Wolfe and Dutton, 2015) may permit the composition and dynamics of the microbiota of grapes from various *Vitis* species to be explored, offering more detailed information on microbial diversity than is retrieved from culture-based studies (Bokulich et al., 2014; Pinto et al., 2014; Setati et al., 2015).

This work reports a detailed analysis on the identity and dynamics of the yeast community present during spontaneous fermentation of grape must from a *Vitis* cultivar different from *Vitis vinifera* L. The collection of strains isolated in this work constitutes a potential source of valuable non-*Saccharomyces* and *S. cerevisiae* strains for enology research as well as for biological, ecological and evolutionary studies on wine yeasts. The study represents the first characterization of indigenous grapevine yeasts from the province of Córdoba, Argentina.

## Author Contributions

Fundamental contributions to the conception and design of the work (MR, CR, SL, AR); acquisition, analysis and interpretation of data (MR, CR, SL, AR); drafting of the work and revising it critically for intellectual content (MR, AR). All authors approved the final version of the manuscript to be submitted for publication and agreed to be accountable for all aspects of the work in ensuring that questions related to the accuracy and integrity of any part of the work are appropriately investigated and resolved.

## Conflict of Interest Statement

The authors declare that the research was conducted in the absence of any commercial or financial relationships that could be construed as a potential conflict of interest.
